# The Unifying Hypothesis of Alzheimer’s Disease: Heparan Sulfate Proteoglycans/Glycosaminoglycans Are Key as First Hypothesized Over 30 Years Ago

**DOI:** 10.3389/fnagi.2021.710683

**Published:** 2021-10-04

**Authors:** Alan David Snow, Joel A. Cummings, Thomas Lake

**Affiliations:** Cognitive Clarity Inc., Edmonds, WA, United States

**Keywords:** Alzheimer’s disease, heparan sulfate, proteoglycans (PGs), glycosaminoglycans (GAGs), mucopolysaccharidosis (MPS), amyloid plaques, neurofibrillary tangles (NFT), inflammation

## Abstract

The updated “Unifying Hypothesis of Alzheimer’s disease” (AD) is described that links all the observed neuropathology in AD brain (i.e., plaques, tangles, and cerebrovascular amyloid deposits), as well as inflammation, genetic factors (involving ApoE), “AD-in-a-Dish” studies, beta-amyloid protein (Aβ) as a microbial peptide; and theories that bacteria, gut microflora, gingivitis and viruses all play a role in the cause of AD. The common link is the early accumulation of heparan sulfate proteoglycans (HSPGs) and heparan sulfate glycosaminoglycans (GAGs). HS GAG accumulation and/or decreased HS GAG degradation is postulated to be the key initiating event. HS GAGs and highly sulfated macromolecules induce Aβ 1–40 (but not 1–42) to form spherical congophilic maltese-cross star-like amyloid core deposits identical to those in the AD brain. Heparin/HS also induces tau protein to form paired helical filaments (PHFs). Increased sulfation and/or decreased degradation of HSPGs and HS GAGs that occur due to brain aging leads to the formation of plaques and tangles in AD brain. Knockout of HS genes markedly reduce the accumulation of Aβ fibrils in the brain demonstrating that HS GAGs are key. Bacteria and viruses all use cell surface HS GAGs for entry into cells, including SARS-CoV-2. Bacteria and viruses cause HS GAGs to rapidly increase to cause near-immediate aggregation of Aβ fibrils. “AD-in-a-dish” studies use “Matrigel” as the underlying scaffold that spontaneously causes plaque, and then tangle formation in a dish. Matrigel mostly contains large amounts of perlecan, the same specific HSPG implicated in AD and amyloid disorders. Mucopolysaccharidoses caused by lack of specific HS GAG enzymes lead to massive accumulation of HS in lysosomal compartments in neurons and contribute to cognitive impairment in children. Neurons full of HS demonstrate marked accumulation and fibrillization of Aβ, tau, α-synuclein, and prion protein (PrP) in mucopolysaccharidosis animal models demonstrating that HS GAG accumulation is a precursor to Aβ accumulation in neurons. Brain aging leads to changes in HSPGs, including newly identified splice variants leading to increased HS GAG sulfation in the AD brain. All of these events lead to the new “Unifying Hypothesis of Alzheimer’s disease” that further implicates HSPGs /HS GAGs as key (as first hypothesized by Snow and Wight in 1989).

## Introduction

This will review the current status of heparan sulfate proteoglycans (HSPGs) and HS glycosaminoglycans (GAGs) in the pathogenesis of AD and related protein misfolded disorders (i.e., amyloid diseases). New evidence further implicates that the Unifying Hypothesis of AD involves HSPGs and HS GAGs as first hypothesized by Snow and Wight in 1989. New studies now implicate these macromolecules further and account for initiating events pertaining to AD pathology (plaques, tangles, cerebrovascular amyloid deposits); as well as unifying many different mechanisms that are all hypothesized to lead to AD and its pathology. We also present an updated model for the specific roles of HSPGs and HS GAGs in initiation and progression of AD based on recent studies and data.

### What Are Proteoglycans (PGs) and Glycosaminoglycans (GAGs)?

Proteoglycans (PGs) are a group of complex macromolecules found in all organs and tissues, intracellularly in a variety of different cell types, or extracellularly in the matrix where they are exported for a variety of functions (Hascall and Hascall, 1981; Gallagher et al., 1986; Poole, 1986; Ruoslahti, 1988). Proteoglycans consist of a linear protein core backbone to which one or more glycosaminoglycan (GAG) chains are covalently linked (Hascall and Hascall, 1981; Hassell et al., 1985). The highly anionic GAG chains consist of repeating disaccharide units containing: (1) hexosamine (either D-glucosamine or D-Galactosamine); and (2) hexuronic acid (either D-glucuronic acid or L-iduronic acid). Several major GAGs have been identified and these include (in order of increased HS GAG sulfation) hyaluronic acid (non-sulfated; in synovial fluid and vitreous humor), chondroitin-4-sulfate (cartilage, bone, heart, brain), chondroitin-6-sulfate (cartilage, bone, heart, brain), dermatan sulfate (skin, blood vessels, heart valves, collagen fibrils), heparan sulfate (basement membranes, blood vessels in body, cell surfaces, brain), heparin (intracellular granules in mast cells lining the arteries in lungs, liver, and skin) and keratan sulfate (cartilage, cornea, bone, brain). Usually, the linkage between the GAG chains and the protein core backbone of a PG consists of a xylose-galactose-galactose attachment region with the xylose molecule covalently linked to the hydroxyl group of a serine residue on the protein core (i.e., Ser-Gly sites; Roden and Armond, 1966). The exception is hyaluronic acid which has a backbone consisting of alternating D-glucuronic acid and D-glucosamine units.

A number of cDNAs encoding the core protein of PGs have been cloned and the partial or complete amino acid sequences have been deduced in the 1980s (Bourdon et al., 1985; Doedge et al., 1986; Day et al., 1987). In the 1990s many new brain HSPGs and other PGs were identified and cloned including perlecan (Noonan et al., 1991), glypicans 1–6 (Karthikeyan et al., 1992), agrin (Tsen et al., 1995; Groffen et al., 1998), syndecans 1–4 (Saunders et al., 1989; Bernfield et al., 1993; Berndt et al., 2001), brevican (Yamada et al., 1994), neurocan (Rauch et al., 1992), and phosphacan (Maurel et al., 1994). Each type of HSPG contains a defined number of HS chains linked to a core protein domain, with syndecans carrying up to five HS GAG chains, while glypicans and secreted HSPGs (i.e., perlecan) usually comprised up to three GAG chains.

Although the PG core protein differs greatly, general features are observed. One such feature is that Ser-Gly regions in the protein core of the PG are potential binding sites for GAG chains (Bourdon et al., 1986; Krusius et al., 1987; Oldberg et al., 1987). Four major classes of PGs, in addition to hyaluronic acid, can be found in most tissues. These include chondroitin sulfate proteoglycans (CSPGs), dermatan sulfate proteoglycans (DSPGs), keratan sulfate proteoglycans (KSPGs), and heparan sulfate proteoglycans (HSPGs). Usually, specific types of PGs predominate in certain tissues and this may relate to specific functional roles these PGs may have in these tissues. HSPGs (like perlecan), a normal component of the basement membranes, may play a role in selective filtration of macromolecules (like in the kidney) and may also serve as a structural organizer of components including collagen, laminin, and fibronectin (Hedman et al., 1982; Koda and Bernfield, 1984; Hassell et al., 1986). Additionally, HSPGs present on the cell surface may mediate cell attachment to a variety of molecules involved in cell adhesion, cell signaling, and neuronal development (Hedman et al., 1982; Koda and Bernfield, 1984). HSPGs are also regulators of axon guidance and synaptic connectivity and specificity (Condomitti and de Wit, 2018).

A number of investigations showed that distinct changes occur in the type and quantity of GAGs and PGs in the brain during development and aging (Aquino et al., 1984; Oohira et al., 1986; Jenkins and Bachelard, 1988). The most interesting thing about PGs and GAGs in the brain and in other tissues is that PG core proteins usually remain the same, whereas GAGs tend to change sulfation patterns and GAG length according to the environment. Thus, GAG chains and their overall sulfation pattern are not static and reflect an ever-changing moving environment in which the GAGs change from one instance to another.

### Highly Sulfated Glycosaminoglycans (GAGs) Are a Common Constituent of All Amyloids

Amyloid diseases are misfolding protein disorders that include the misfolding of beta-amyloid protein (Aβ) into fibrillar deposits in amyloid plaques and cerebrovascular amyloid deposits in Alzheimer’s disease (AD). Even tau protein, a normal microtubule binding protein, becomes altered inside cells and leads to the abnormal formation of paired helical filaments as part of neurofibrillary tangles (NFTs). Amyloid diseases refer to a number of unique proteins (including the beta-amyloid protein or Aβ of AD) that all share a common misfolding problem. About 15 different amyloid disorders have been identified that are characterized by a similar fibrillar end product in tissues consisting of a predominant beta-sheet secondary structure positively stained with Congo red (i.e., with a characteristic red/green birefringence when viewed under polarized light; Puchtler et al., 1962) and fibrils of 7–10 nm in diameter when observed at the electron microscopic level (Snow and Wight, 1989). Different amyloid proteins include AA amyloid (inflammation-associated), AL amyloid, Beta_2_-microglobulin, senile cardiac amyloid (prealbumin), beta-amyloid protein or Aβ of Alzheimer’s disease (AD) and Down’s syndrome, and prion diseases with PrP proteins [(including Creutzfeldt-Jakob disease (CJD), Gerstmann-Straussler syndrome (GSS), kuru and animal scrapie] all misfold into beta-sheet insoluble fibrillar deposits (reviewed in Snow and Wight, 1989; Snow and Castillo, 1997).

### Heparan Sulfate Glycosaminoglycans (GAGs) Accumulate Concurrent or Prior to AA Amyloid Deposits Are Observed in Tissues

**Figure 1:**
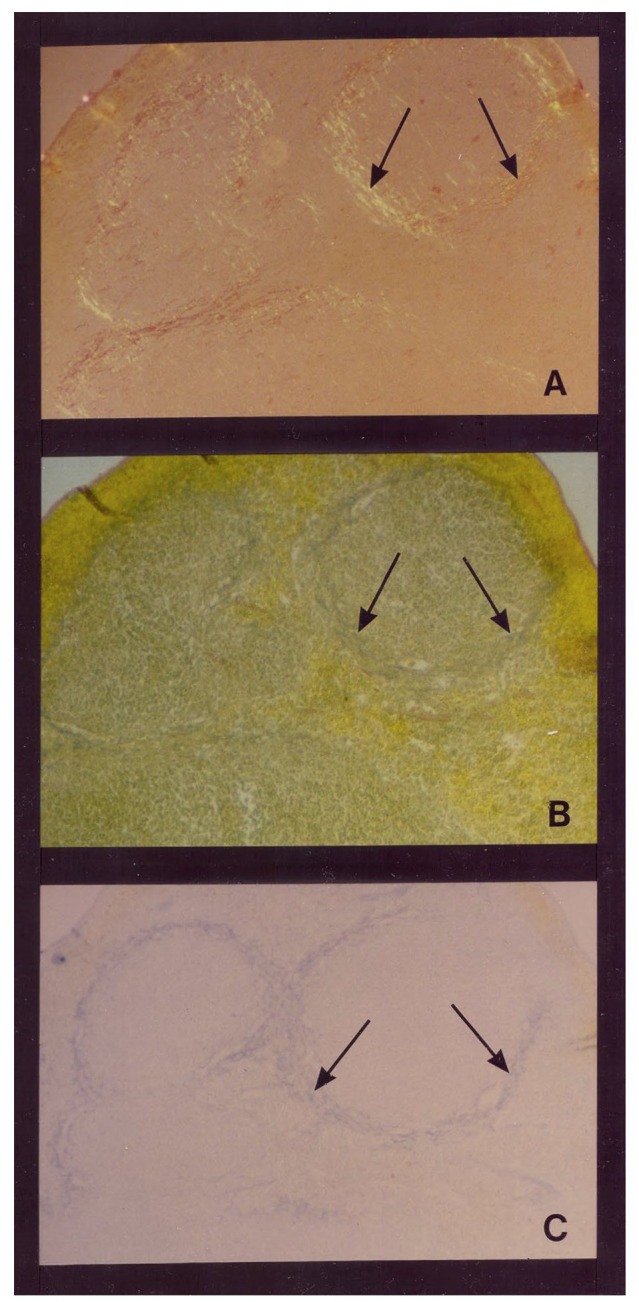
Temporal relationship between AA amyloid deposition and heparan sulfate glycosaminoglycan (GAG) accumulation in the spleen in a mouse model of experimental AA amyloidosis. AA amyloid deposition and sulfated GAG deposition in the perifollicular areas of spleen at 48 h post-induction (with amyloid enhancing factor + silver nitrate (Snow, 1986). **(A)** Splenic amyloid (arrowheads) has extended to most perifollicular areas. Congo red staining for amyloid deposits under polarized light. **(B)** Positive Sulfated Alcian-Blue (SAB; arrowheads) seen in the exact perifollicular areas as amyloid deposits. **(C)** Positive Alcian blue staining at 0.7 M magnesium chloride demonstrating accumulation of primarily highly sulfated GAGs at amyloid deposition (Snow, 1986).

Snow et al. (Snow and Kisilevsky, 1986; Snow, 1986) first demonstrated that highly sulfated GAGs accumulate early and in the same exact tissue locations (i.e., in the perifollicular area of the spleen; central veins in the liver and proximal convoluted tubules in the kidney) of fibrillar AA amyloid deposits in tissues using an experimental mouse model of AA (inflammation-associated) amyloidosis that was induced by amyloid enhancing factor (AEF) and an inflammatory stimulus (i.e., silver nitrate). This was an important model because for the first time one can slowly watch amyloid fibril formation in tissues and correlate it with highly sulfated GAG accumulation in tissues as well. At 36 h, the first wisps of splenic amyloid were detected in the perifollicular area of the spleen (as shown by positive Congo red staining; Snow, 1986). In the same 36-h time period and in the exact same location (i.e., perifollicular area of the spleen), sulfated GAGs were identified as shown by positive sulfated Alcian-blue (SAB) green staining (indicated of sulfated GAGs; Scott and Dorling, 1965; Dorling, 1969) and Alcian blue staining with 0.3 M and 0.7 M magnesium chloride (Dorling, 1969). These studies demonstrated that initial deposition of AA amyloid fibrils in tissues (spleen at 36–48 h; liver at 2–3 days and kidney at 7 days) in a mouse model was essentially concurrent and in the exact tissue locations as the initial accumulation of highly sulfated GAGs. Deposition at 48 h post-induction (AEF + silver nitrate) showed an extension of both AA amyloid deposits and highly sulfated GAGs (Figures 1A–C; Snow, 1986). These studies initially suggested that highly sulfated HS GAGs and HSPGs accumulate early and in the same tissue locations as AA amyloid deposits in an animal model of experimental AA amyloidosis (Snow, 1986). Snow et al. (1990a) later demonstrated that highly sulfated proteoglycans accumulated exactly in tissue where AA amyloid fibrils were deposited by using Ruthenium red and Cuprolinic blue staining to visualize PGs at the electron microscopic (EM) level in association with AA amyloid fibrils. PGs are usually washed out during normal EM fixation and the use of Ruthenium red and Cuprolinic blue allowed retention and visualization of PGs. Using newly available antibodies to the core protein or HS GAG chains of the HSPG known as perlecan, demonstrated a virtually concurrent deposition of perlecan and AA amyloid in specific tissue sites regardless of the organ involved (spleen or liver) or the induction protocol used (amyloid enhancing factor + silver nitrate, or daily azocasein injections; Snow et al., 1990a). Immunogold labeling of HSPGs in amyloidotic mouse spleen or liver revealed specific co-localization of HSPGs to amyloid fibrils (Snow et al., 1990a; Snow, 1991).

A few years later using the same AA amyloid mouse model, Ailes et al. (1993) made the startling discovery that perlecan gene expression increased prior to AA amyloid fibril deposition in tissues. A two-fold induction of perlecan mRNA occurred 24-h post-stimulation, a time when AA amyloid fibrils were not detectable in tissues, either by staining (i.e., Congo red) or by immunohistochemistry. The induction of perlecan gene expression occurring before the onset of amyloid fibril formation supported a role for perlecan and HSPGs in the initiating stages of amyloid fibril formation.

### Heparan Sulfate Proteoglycans (HSPGs): A Common Constituent of All Amyloid Diseases

In the late 1980s and early 1990s specific antibodies to heparan sulfate proteoglycans (HSPGs) were being developed and unique staining methods included the sulfated Alcian blue (SAB) method of Scott and Dorling (1965), and the Alcian blue magnesium chloride method of Dorling (1969), allowed one to first visualize highly sulfated GAGs in tissues. Initial studies by Snow et al. (1987a) demonstrated that sulfated GAGs were a common constituent of all amyloids independent of the amyloid protein deposited. Comprehensive studies indicated that all ~15 amyloid disorders with different amyloid proteins all demonstrated the presence of highly sulfated GAGs and HSPGs colocalized to the amyloid fibril deposits suggesting that HSPGs probably played an important role in the pathogenesis of amyloid diseases (Snow et al., 1987b; Young et al., 1989).

### Isolation of Perlecan: A Specific Heparan Sulfate Proteoglycan (HSPG) From Engelbreth-Holm-Swarm (EHS) Sarcoma

**Figure 2:**
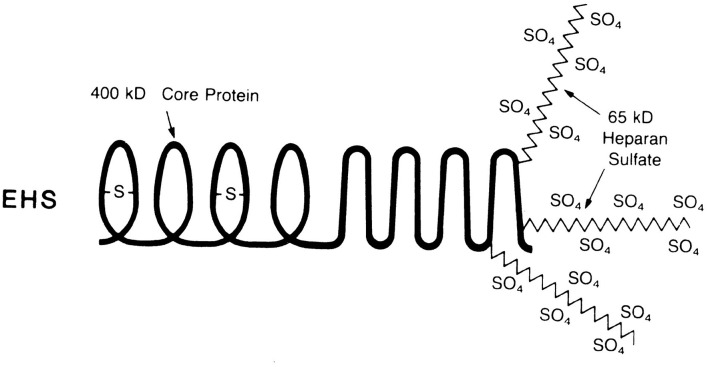
Structure of the heparan sulfate proteoglycan perlecan derived from the Engelbreth-Holm-Swarm (EHS) sarcoma. The perlecan proteoglycan has a core protein of 400,000 MW containing three heparan sulfate chains of 65,000 MW each (Snow and Wight, 1989).

The specific HSPG, perlecan, was cloned and sequenced, and later found to be important for understanding the pathological consequences of amyloid deposition (Klein et al., 1988). In addition, new research into the effects of heparan sulfate (GAGs) vs. less sulfated GAGs were being tested. Figure 2 shows the structure of perlecan which consists of a 400,000 MW core protein containing three heparan sulfate chains of 65,000 MW each (Hassell et al., 1985; Kato et al., 1988). Polyclonal (Hassell et al., 1980) and monoclonal antibodies (Kato et al., 1988) to the HSPG core protein immunolocalized HSPGs to the neuritic plaques and cerebrovascular amyloid deposits in the brains of Alzheimer’s patients (Snow et al., 1988a), as described below. Perlecan was isolated by Castillo et al. (1998) and tested in a variety of different studies to assess its effects on amyloid deposition including the first model to mimic fibrillar Aβ in the brain (Snow et al., 1994a) before any transgenic models for amyloid plaque deposition in the brain were initially discovered.

### Heparan Sulfate Proteoglycans (HSPGs) and Heparan Sulfate Glycosaminoglycans (GAGs) Are Also Localized to the Pathological Lesions in Alzheimer’s Disease, Down’s Syndrome, and Prion Diseases

The pathological hallmark of AD includes the presence in the brain of extracellular amyloid plaques (consisting of the 42-amino acid beta-amyloid protein or Aβ; Glenner and Wong, 1984; Masters et al., 1985), neurofibrillary tangles (filaments inside neurons consisting of tau protein; Iqbal et al., 1986; Kosik et al., 1986; Lee et al., 1991), and cerebrovascular amyloid deposits in the walls of blood vessels (containing Aβ 1–40 and 1–42; Pardridge et al., 1987). Amyloid plaques consist of star-shaped amyloid fibrils seen at the electron microscopic level with neurites within, whereas tangles consist of tau in the form of paired helical filaments, as well as straight filaments (Iqbal et al., 1986; Kosik et al., 1986; Lee et al., 1991). In addition, in the late stages of AD marked inflammation contributes to the decline in patients to memory loss and eventual dementia.

**Figure 3:**
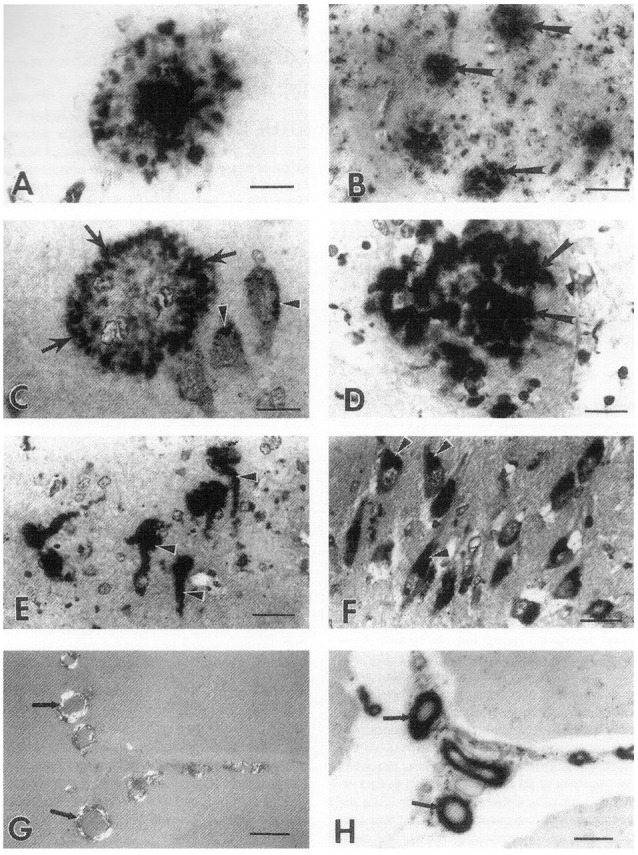
Immunolocalization of heparan sulfate GAGs in neurons and in the characteristic lesions of AD. **(A)** immunostaining of an amyloid plaque in the hippocampus of a 90-year old woman with AD (obtained 5.5 h after death using the monoclonal antibody (HK-249) recognizing HS GAG chains. Bar, 21 μm. **(B)** Immunostaining of primitive plaques (arrows) in the amygdala of a 64-year old man with AD (4 h after death). Bar, 80 μm. **(C)** Heparan sulfate GAG immunostaining (HK-249) in the periphery (arrows) of a primitive plaque in the hippocampus of a 90-year old woman with AD (5 h after death). Note HS immunostaining also in the cell bodies of adjacent neurons (arrowheads). Bar, 20 μm. **(D)** Strong immunostaining of a primitive plaque using HK-249 antibody in the hippocampus of a 67-year old man with AD (5.5 h after death). Note immunostaining of neurites in plaque (arrows). Bar, 20 μm. **(E)** Heparan sulfate GAG immunostaining (HK-249) of neurofibrillary tangles (arrowheads) in the amygdala of a 64-year old man with AD (4 h after death). Bar, 40 μm. **(F)** Strong immunostaining in the cell bodies of neurons (arrowheads) using the HK-249 HS GAG chain antibody in the hippocampus of a 90-year old woman with AD (5.5 h after death). These neurons do not contain tangles because on adjacent serial sections they were negative for Congo red staining, Bar, 40 μm. **(G)** Positive Congo red staining of meningeal blood vessels (arrows) in the cerebellum of a 74-year old man with AD indicating the presence of cerebrovascular amyloid in these vessels, Bar, 108 μm. **(H)** Serial section of tissue shown in **(G)** demonstrates HK-249 HS GAG immunostaining in meningeal blood vessels containing amyloid deposits (arrows). Bar, 108 μm (Snow et al., 1990b).

An early study (Suzuki et al., 1965) suggested that AD brain tissue obtained at autopsy contained an increased amount of GAGs (determined by hexuronic acid analysis) compared to those present in the AD brain. Histochemical techniques were used to demonstrate highly sulfated GAGs and HSPGs present in all the characteristic lesions of AD including neuritic and amyloid plaques, neurofibrillary tangles, and cerebrovascular amyloid deposits (Snow and Wight, 1989; Snow et al., 1989b, 1990b, 1994b; Perry et al., 1990, 1991). Initial studies used sulfated-Alcian blue (SAB) staining to demonstrate sulfated GAGs in neuritic plaques were also immunostained with Aβ antibodies (Snow and Wight, 1989). With the development of a specific HS GAG chain antibody (known as HK-249), HS GAGs were specifically immunolocalized to neurons (Figures 3E,F), amyloid plaques (and neuritic plaques; Figures 3A–D), and cerebrovascular amyloid deposits in the AD brain (Figures 3G,H; Snow et al., 1990b). Others showed accumulation of HSPGs and HS GAGs in the neurofibrillary tangles of AD (Perry et al., 1990, 1991). HS GAGs also were immunolocalized in the Aβ plaques in Down’s syndrome brain (i.e., trisomy 21 that produced similar Aβ accumulation to that seen in AD brain but at a much earlier age; Snow et al., 1990b). Pyramidal and granule cells contained neurons markedly immunostained with HS GAG chain antibodies (HK-249) even before any amyloid plaques or neurofibrillary tangles were observed (Snow et al., 1990b). Conformation of the positive HS GAG chain antibody specificity was observed following pre-absorption with HSPG antigen (Snow et al., 1990b). Heparan sulfate GAGs accumulated in Down’s syndrome brain as early as 1 day after birth in pyramidal neurons in the hippocampus before any observed Aβ accumulation in neurons further confirming that HS GAG accumulation is an initiating event and occurs prior to Aβ accumulation (Snow et al., 1990b).

**Figure 4:**
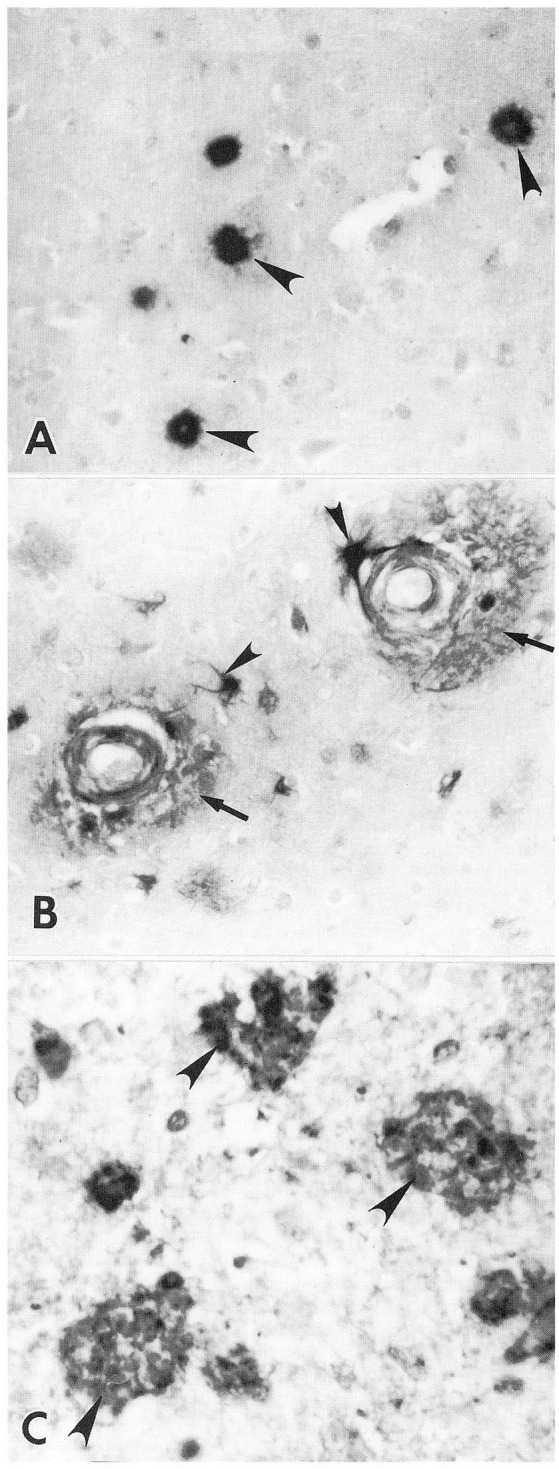
Immunolocalization of HSPG core protein in AD brain. **(A)** Positive immunostaining of plaque amyloid (arrowheads) with a monoclonal antibody (HK-102) to the core protein of HSPGs in a 72-year old man with AD. Original mag. ×276. **(B)** Positive anti-HSPG immunostaining of cerebrovascular amyloid deposits (arrows) in AD brain of a 92-year old female patient. Note positive staining of adjacent astrocytes (arrowheads) Original mag. ×276. **(C)** Positive immunostaining of primitive plaques (arrowheads) with anti-HSPG polyclonal antibody in a 92-year old female patient with AD. Original mag. ×616 (Snow and Wight, 1989).

Heparan sulfate GAGs were also present in amyloid plaques in prion (PrP protein) diseases including Creutzfeldt-Jakob disease (CJD), Gerstmann-Straussler syndrome (GSS), kuru, and animal scrapie (Snow and Wight, 1989; Snow et al., 1989a, 1990c). Co-localization of highly sulfated GAGs and PrP protein was demonstrated in all these diseases. In a unique case of familial GSS multi-core amyloid plaques in the cerebral cortex of a 61-year-old man showed co-localization of PrP protein and highly sulfated GAGs (Nochlin et al., 1989). HSPGs (using a newly developed perlecan core protein antibody known as HK-102) demonstrated HSPGs (i.e., perlecan) immunolocalized to amyloid plaques, neuritic plaques, and astrocytes in the human AD brain (Figure 4; Snow et al., 1988a).

**Figure 5:**
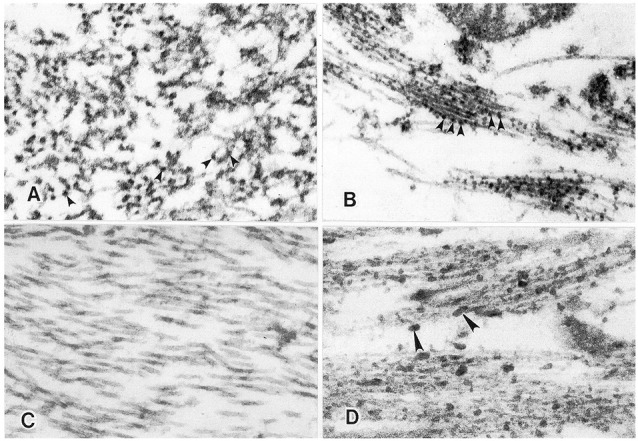
Proteoglycans visualized in neuritic plaques and neurofibrillary tangles as demonstrated by Ruthenium red and Cuprolinic blue staining. **(A)** Ruthenium red staining of neuritic plaque in the hippocampus of a 92-year old female with AD. Positive Ruthenium red granules (arrowheads) are specifically localized along amyloid fibrils present with the neuritic plaque. **(B)** Ruthenium red staining of paired helical and straight filaments in a neurofibrillary tangle in the hippocampus of a 72-year old male with AD. Positive Ruthenium red granules are observed (arrowheads) specifically localized to the paired helical and straight filaments. Orig. Mag., ×80,000. **(C)** In the absence of Cuprolinic blue, no apparent staining of paired helical and straight filaments in a neurofibrillary tangle is observed in the hippocampus of a 92-year old female with AD. Original mag. ×80,000. **(D)** In the presence of Cuprolinic blue at 0.3 M magnesium chloride, proteoglycans are visualized as positive Cuprolinic blue filaments (arrowheads) in intimate association with bundles of paired helical and straight filaments in a neurofibrillary tangle in the hippocampus of a 92-year old female with AD. Original Mag. ×80,000 (Snow et al., 1989b).

Proteoglycans, which are usually washed out during electron microscopy processing, can be retained in tissues using specific staining techniques including Ruthenium red and Cuprolinic blue (Scott and Dorling, 1965; Dorling, 1969). For the first time, an exact colocalization of PGs (seen as granules with the GAG chains condensed onto the protein core) was observed in amyloid plaques (Figure 5A) and neurofibrillary tangles (Figure 5B) using the cationic dye Ruthenium red (Snow et al., 1989b). Cuprolinic blue staining at 0.3 M magnesium chloride also demonstrated PGs specifically localized with a periodicity to the paired helical and straight filaments in tangles (seen as short filaments with the GAG chains condensed onto its core proteins; Figure 5B; Snow et al., 1989b). In the absence of Curprolinic blue, no apparent staining of PGs was observed in neurofibrillary tangles as shown in Figure 5C. These studies demonstrated the close ultrastructural relationship between HSPGs and amyloid plaques and neurofibrillary tangles (i.e., the characteristic pathological lesions of AD).

Later, inflammatory cytokine producers, astrocytes and microglia also were shown to manufacture HSPGs as part of the inflammation process which also has been implicated in AD (Miller et al., 1997). HSPGs were also present in amyloid plaque cores isolated from the AD brain (Selkoe et al., 1986; Snow and Wight, 1989) further demonstrating HSPGs as an integral part of amyloid plaques in AD brain.

Heparan sulfate GAG chains (HK-249 antibody) and core protein (HK-102 antibody) were further immunolocalized to amyloid core plaques, neuritic plaques, and diffuse plaques in the AD brain (Snow et al., 1988a; Snow et al., 1990b). Cerebellar diffuse amyloid plaques that did not contain HS GAG staining never demonstrated Aβ deposits in a fibril form further implicating that HS GAG presence is needed to form fibrillar Aβ deposits as seen in the hippocampus and cortex of AD (Snow et al., 1994b). Both Bielchowsky silver staining and Aβ 1–40 immunostaining, but lack of HS GAG staining in cerebellar plaques, but not blood vessels, or choroid plexus basement membrane suggested that HS GAGs were the missing piece needed for fibrillar Aβ fibrillar formation in tissues (Snow et al., 1994b). This was confirmed in cases of mucopolysaccharidosis (MPS), whereby HS GAG accumulation in neurons lead to progressive aggregation of Aβ, tau, α-synuclein, and prion proteins in neurons in brains of young children with HS enzyme deficiencies (i.e., lack of sulfatases to degrade the accumulation of HS GAGs; Ginsberg et al., 1999; Reinhard et al., 2013; Beard et al., 2017; Bigger et al., 2018).

Other HSPGs later found to be present in amyloid and neuritic plaques and elevated in AD brain (Liu et al., 2016) besides perlecan included the HSPGs agrin, glypican-1, glypican-3, and syndecans 1–4 (Verbeek et al., 1999; Watanabe et al., 2004; Liu et al., 2016; Lorenta-Gea et al., 2020). Other PGs identified in AD brain lesions included dermatan sulfate proteoglycans (Snow et al., 1992) and keratan sulfate proteoglycans (Snow et al., 1996) However, it appears that HSPGs play the most important role in the pathogenesis of AD and other misfolded protein disorders.

### The Sulfate Moieties on HS GAGs Are Essential for Enhancement of Aβ 1–40 Fibril Formation

Castillo et al. (1999) demonstrated that the sulfate moieties on HS GAGs were critical for the enhancing effects of HS GAGs on Aβ 1–40 fibril formation. Sulfated GAGs including heparin, heparan sulfate, dextran sulfate, pentosan sulfate, and to a lesser extent chondroitin-4-sulfate significantly enhanced Aβ 1–40 peptide to form amyloid fibrils (as measured by Thioflavin T fluorometry) much faster (almost immediately) than with Aβ 1–40 alone. The sulfate moieties of GAGs were essential for the observed effect since the removal of all sulfates from heparin (i.e., completely desulfated N-acetylated heparin) led to a complete loss in the enhancement of Aβ fibrillogenesis (Castillo et al., 1999). On the other hand, removal of O-sulfate from heparin (i.e., completely desulfated N-sulfated heparin) and to a lesser extent N-sulfate (i.e., N-desulfated N-acetylated heparin) resulted only in a partial loss of enhancement of Aβ 1–40 fibril formation (Castillo et al., 1999).

### Perlecan Infused Into Rat Hippocampus With Aβ 1–40 Leads to Persistence of Aβ Fibrils in Brain

Continuous infusion of Aβ 1–40 + perlecan (specific HSPG) into rat brain led to one of the first animal models for amyloid plaque persistence and stability in rat brain (Snow et al., 1994a). This was before any transgenic mice for beta-amyloid precursor protein were ever developed. Positive Congo red staining, Aβ immunostaining, perlecan immunostaining, and Thioflavin S fluorescence all showed continued co-localization of HSPGs to Aβ fibril deposits (Snow et al., 1994a). Maltese-cross spherical congophilic amyloid plaques that demonstrated the classic red/green birefringence identical to amyloid plaques observed in AD brain suggested that HSPGs may induce Aβ 1–40 to spontaneously form amyloid plaques (Snow et al., 1994a).

### Heparan Sulfate GAGs Key to Amyloid Plaque Core Formation Identical to That Observed in AD

**Figure 6:**
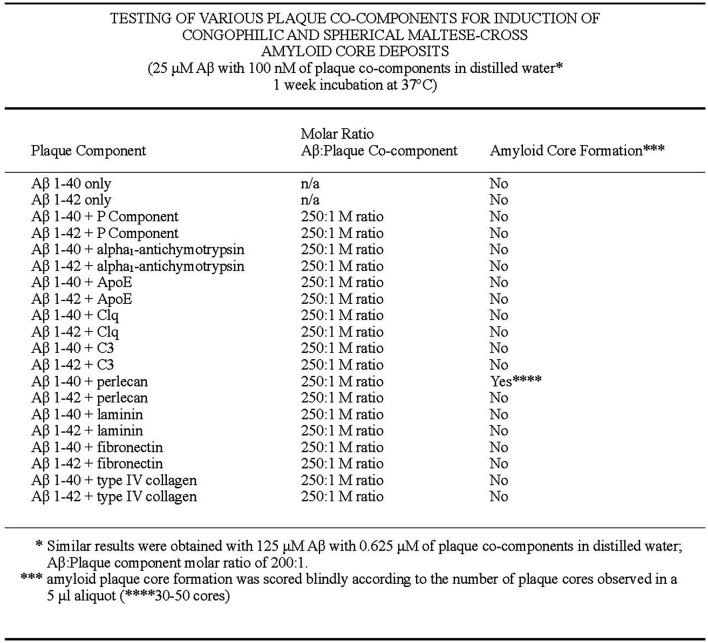
Testing of various plaque core components for induction of congophilic and spherical maltese-cross amyloid core deposits. Different known amyloid plaque co-components including P component, alpha_1_-antichymotrypsin, ApoE, C1q, C3, perlecan (HSPG), laminin, fibronectin, and type IV collagen were tested for their ability to form congophilic spherical maltese-cross amyloid core deposits. Only perlecan (HSPG) were shown to induce congophilic spherical maltese-cross amyloid core deposits identical to those seen in the AD brain (Castillo and Snow, 2006).

**Figure 7:**
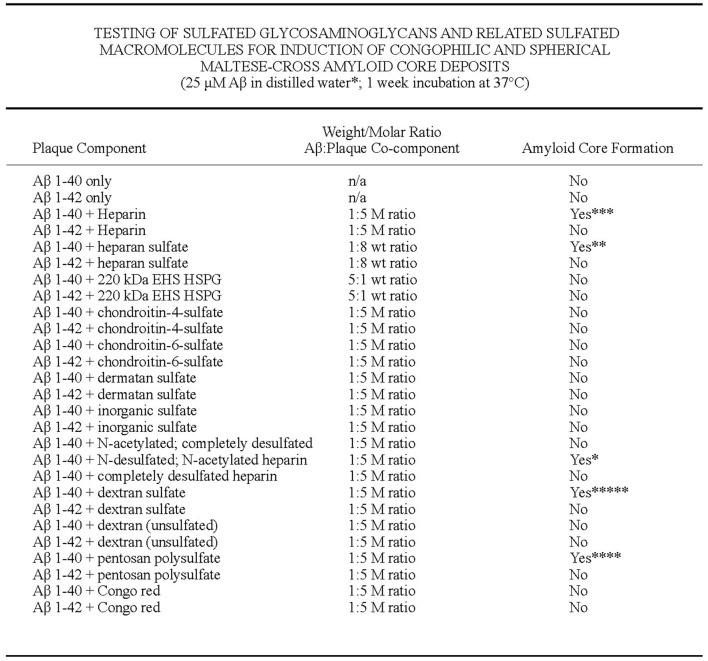
Testing of sulfated GAGs and related sulfated macromolecules for induction of congophilic and spherical maltese-cross amyloid core deposits. Aβ 1–40 and Aβ 1–42 in the absence of the presence of different sulfated GAGs and related macromolecules were tested to induce congophilic spherical maltese-cross amyloid core deposits as those found in AD brain. Only highly sulfated GAGs and related macromolecules including heparin, heparan sulfate, dextran sulfate, pentosan polysulfate, and perlecan (isolated from Engelbreth-Holm-Swarm sarcoma) induced Aβ 1–40 (but not Aβ 1–42) to form congophilic spherical maltese-cross amyloid core deposits identical to those seen in the human AD brain. The preferred molar: ratio of Aβ:sulfated GAG ratio was about 1:5 (with Aβ 1–40 at 25 μM). On the other hand, the preferred weight ratio of Aβ: heparan sulfate for congophilic maltese-cross amyloid plaque formation was 1:8 (i.e., 50 μg Aβ 1–40 in 100 μl of distilled water with 400 μg of heparan sulfate). Congophilic maltese-cross amyloid core formation under the same molar ratios were not observed for chondroitin-4-sulfate, chondroitin-6-sulfate, dermatan sulfate, inorganic sulfate, N-acetylated completely desulfated heparin, and unsulfated dextran indicating that highly sulfated GAGs and related macromolecules were primarily needed for induction of amyloid plaque core formation. Only Aβ 1–40, but not Aβ 1–42 induced amyloid plaque core formation with highly sulfated GAGs, perlecan and highly sulfated macromolecules [(including dextran sulfate, pentosan polysulfate, and polyvinyl sulphonate (Castillo and Snow, 2006). Amyloid plaque core formation was scored blindly according to the number of plaque cores observed in a 5 μl aliquot (*1–5 cores, **5–10 cores, ***10–30 cores, ****30–50 cores, *****50 cores).

**Figure 8:**
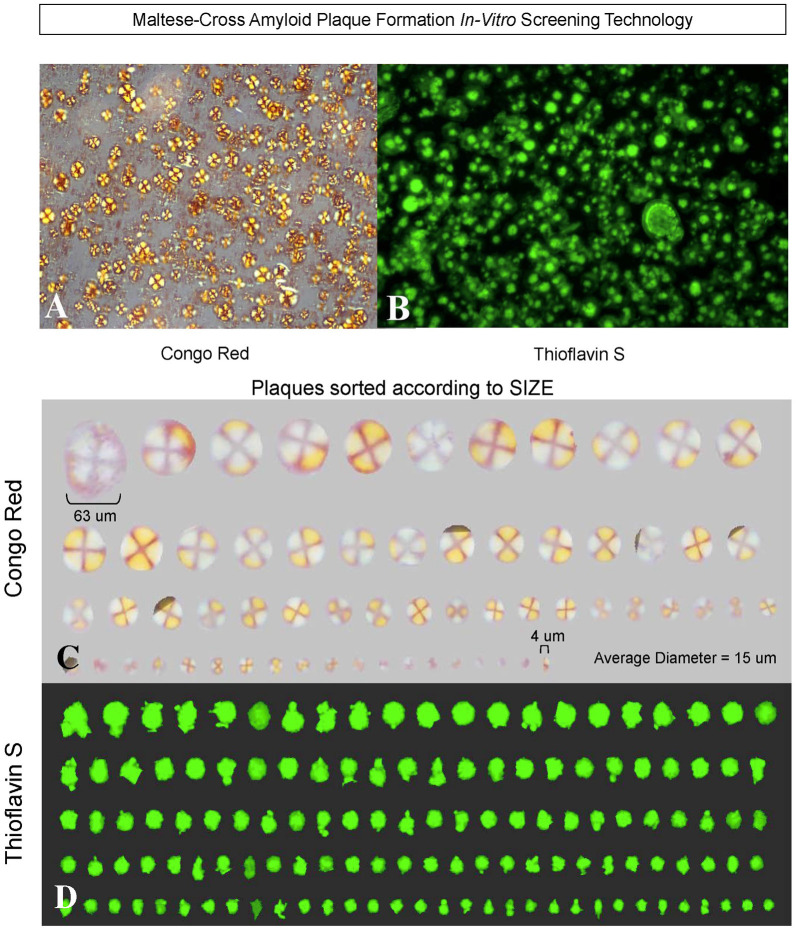
Heparan sulfate GAGs induce Aβ 1–40 (but not Aβ 1–42) to form spherical congophilic and thioflavin S maltese-cross amyloid plaques identical to those seen in AD brain. Heparan sulfate GAGs + Aβ 1–40 (but not Aβ 1–42) at 1:8 molar ratio (i.e., 50 μg Aβ 1–40 in 100 μl of distilled water with 400 μg of heparan sulfate) spontaneously form (within minutes) spherical congophilic maltese-cross amyloid plaques **(A)** and Thioflavin S plaques **(B)** identical to those observed in AD brain. Spherical maltese-cross amyloid cores were not observed with Aβ 1–40 or Aβ 1–42 alone. **(C)** Congo red plaques formed by Aβ 1–40 + heparan sulfate GAGs sorted according to size, ranging from 4 μm up to 63 μm, with an average diameter of 15 μm. Note the red-green birefringence of the amyloid cores as viewed under polarized light. Rotation of the polarizer changes the red color to green, and the green color to red (i.e., red/green birefringence). This also occurs identically in the human AD brain following staining with Congo red and viewing brain sections under polarized light. **(D)** Thioflavin S fluorescence demonstrating the various sizes of amyloid cores formed after incubation of Aβ 1–40 + heparan sulfate GAGs (Castillo and Snow, 2006).

The first formation of congophilic spherical maltese-cross amyloid stars (with a red/green birefringence under polarized light) was discovered by Castillo and Snow (2006) to decipher this characteristic pathology observed in all brains of AD. These studies went unnoticed since they were only described in detail in a US patent #7,148,001 B2 (Castillo and Snow, 2006) that was issued over 15 years ago and not published in the broad literature. First, they tested different amyloid plaque co-components for induction of congophilic and spherical maltese-cross amyloid core deposits including testing Aβ 1–40, Aβ 1–42, P component, alpha_1_-antichymotrypsin, ApoE, C1q, C3, and basement membrane components including perlecan, laminin, fibronectin and type IV collagen (Figure 6; Castillo and Snow, 2006). Only perlecan (a specific HSPG) and Aβ 1–40 induced congophilic maltese-cross formation of amyloid core deposits identical to those seen in AD brain. In the next set of studies, highly sulfated GAGs (including heparin, heparan sulfate, perlecan, dextran sulfate, and pentosaulfate) caused the formation of Aβ 1–40 (but not Aβ 1–42) to spontaneously form congophilic spherical maltese-cross deposits (Figure 7; Castillo and Snow, 2006). The preferred molar ratio of Aβ 1–40: sulfated GAG was critical and optimized at about 1:5 (with Aβ 1–40 at 25 μM), with the preferred ratio for Aβ 1–40: heparan sulfate to be 1:8. No maltese-cross amyloid plaque core formation was induced by chondroitin-4-sulfate, chondroitin-6 sulfate, dermatan sulfate, inorganic sulfate, N-acetylated-completely desulfated heparin, completed desulfated N-sulfated heparin, unsulfated dextran, or congo red; Figure 7). These studies demonstrated that HS GAGs are predominately responsible for inducing the observed congophilic maltese-cross star amyloid plaques observed in the human AD brain and that the sulfate groups on HS GAGs are critical (Castillo and Snow, 2006). Figure 8 demonstrates the massive and spontaneous generation of maltese-cross amyloid plaques following a few minutes of co-incubation with Aβ 1–40 + HS GAGs at a 1:8 molar ratio (50 μg of Aβ 1–40 in 100 μl of distilled water with 400 μg HS). Note the massive accumulation of spherical maltese cross amyloid plaques as shown by Congo red staining (Figures 8A–C) and Thioflavin S fluorescence (Figures 8B–D; Castillo and Snow, 2006). The plaque core sizes of congophilic maltese-cross varied from 4 μm to 63 μm with an average diameter of 15 μm (Figure 8C). The induction of Aβ to spontaneously form maltese-cross amyloid cores of varying sizes by HS is exactly what one observes in the AD brain and in brains of animals infused with perlecan and Aβ 1–40 (Snow et al., 1994a), as well in brains of beta-amyloid precursor protein (APP) transgenic mice (Cummings et al., 2002).

A series of articles by the Tanzi lab (Choi et al., 2014; Kim et al., 2015; Jorfi et al., 2018; Park et al., 2018) recapitulated plaques, and then tangles in a cell culture dish and cited this is the first time this phenomenon has spontaneously occurred in their “AD-in-a-dish” phenomenon. However, the key was their continued use of “Matrigel” (that predominantly contains perlecan, the EHS sarcoma HSPG; Castillo et al., 1996) that helps spontaneously form amyloid plaques (Castillo and Snow, 2006). Even in their studies on human “neurospheroid arrays” (Jorfi et al., 2018), they used Matrigel in each well to form spherical arrays of Aβ fibril formation and tau protein tangles. It is postulated that the perlecan in “Matrigel” and its highly sulfated HS chains are important in spontaneous plaque, and then tangle formation (Castillo et al., 1996; discussed further below).

### Perlecan and Its Heparan Sulfate (HS) Chains Accelerate Aβ 1–40 Fibril Formation and Maintain Aβ 1–42 Fibril Stability

Perlecan (a specific HSPG) isolated from EHS tumor defined perlecan interactions with Aβ and its effects on Aβ fibril formation and stability (Castillo et al., 1997). Perlecan accelerated Aβ 1–40 fibril formation within 1-h of interaction and stopped Aβ 1–42 from being degraded over a 2-week period (Castillo et al., 1997). The HS GAG sulfated chains of perlecan were responsible for this activity. These studies demonstrated that HS GAGs in the AD brain likely accelerate Aβ fibril formation and prevent its usual degradation, further implicating HSPGs in the pathogenesis of AD (Castillo et al., 1997).

Differential binding of vascular cell-derived proteoglycans (perlecan, biglycan, decorin, and versican) were compared to their binding with Aβ (Snow et al., 1995). Perlecan bound with high affinity with approximately 1 mole of perlecan binding 1.8 moles of Aβ. Weak binding with decorin and biglycan (two dermatan sulfate PGs), and no binding with versican (large chondroitin sulfate PG) was found. The perlecan HS GAG binding site on Aβ was identified between positive charged residues at resides 13–16 (Snow et al., 1995).

### Heparan Sulfate GAG Accumulation Is Central to Tau Protein Tangle Formation and Brain Spread

Highly sulfated heparin and heparan sulfate GAGs can induce tau protein to form paired helical filaments identical to those observed in AD brain in tangles (Goedert et al., 1996; Pudel and Wei, 1999). This formation appears to be maximized at a 1:1 complex of tau monomer to heparin (Zhu et al., 2010) and studies suggest that the 6-O-sulfate group and a specified length of GAG chain on HSPGs are critical for tau induction (Zhao et al., 2017; Rauch et al., 2018; Stopschinski et al., 2018; Fichou et al., 2019) and internalization. Tau fibrils in neurons occur *via* binding to HSPGs (Holmes et al., 2013). This is blocked in cultures cells and primary neurons by heparin, heparinase, and by a genetic knockdown of a key HSPG synthetic enzyme Ext1 (Holmes et al., 2013). Cells deficient in Ext1 cannot produce HSPGs although they can produce other GAGs. Interference with tau binding to HSPGs prevented recombinant tau fibrils from inducing intracellular aggregation and blocked transcellular aggregate propagation (Holmes et al., 2013; Stopschinski et al., 2018). α-synuclein fibrils (major insoluble beta-sheet protein in Lewy bodies) use the same entry mechanism to seed intracellular aggregation (Holmes et al., 2013). These studies demonstrate that HSPGs are a receptor for cell uptake of both tau and α-synuclein. The spreading of tau from one cell to another also involves the binding of HSPGs on the cell surface making it an important target for AD therapeutics.

### Heparan Sulfate Proteoglycans/Glycosaminoglycans: A Potential Role in the Cell Cycle?

Recent studies suggest that there may be changes in cell cycle reentry of hippocampal and cortical neurons in AD that may precede the observed proteinopathies (i.e., beta-amyloid fibrils and tau tangles) in AD (Arendt et al., 1996; Frade and Ovejero-Benito, 2015; Nativio et al., 2018; Beckmann et al., 2021; Portillo et al., 2021). Analyses suggest that AD may not simply be an advanced state of normal aging, but rather a dysregulation of aging that may induce specific chromatin structural changes and/or transcription programs (Nativio et al., 2018). The mechanistic parallels between tauopathies and cancer are emerging (Beckmann et al., 2021) in which AD and related tauopathies in the neuronal upregulation of proteins are associated with cell cycle activation (Beckmann et al., 2021). The studies by Beckmann et al. (2021) using *Drosophila* suggest that the tau protein is a driver of abortive cell cycle activation in neurons. This hypothesis has been confirmed by a number of earlier studies (Andorfer et al., 2005; McShea et al., 2007; Seward et al., 2013). Studies by Portillo et al. (2021) also recently indicate that SIRT6 may regulate tau protein by directly deacetylating it in the nucleus. Chromatin fraction samples of AD case subjects demonstrated an increase in nuclear tau in general, as well as its acetylated and phosphorylated forms (Portillo et al., 2021). In addition, under stress conditions, like DNA damage or models of accelerated aging, such as in SIRT6-deficient brains, the nuclear localization of tau is increased (Portillo et al., 2021).

Specific HSPGs and HS GAGs have also been shown to play a role in cell cycle reentry and are present in the nucleus of cells (Chen and Sanderson, 2009; Stewart and Sanderson, 2014; Shaberg et al., 2021). Heparanase (implicated in AD and related disorders) regulates levels of the HSPG syndecan-1 in the nucleus (Chen and Sanderson, 2009). The HSPG glypican was discovered in the nucleus of neurons and glioma cells (Liang et al., 1997). Heparan sulfate PGs/ HS GAGs in the nucleus may act to repress transcriptional activity. The role of HS in the nucleus has been linked to control of cell proliferation including the shuttling of the heparin-binding growth factor FGF2, inhibition of DNA topoisomerase I activity, and stabilization of the mitotic machinery (Ferdarko et al., 1989; Liang et al., 1997; Kovalszky et al., 1998; Hsia et al., 2003). The tightly controlled regulation of HSPG and HS trafficking to the nucleus argues they have specific and synchronized regulatory functions.

The composition of HS in the nucleus may dictate whether cell division is stimulated or blocked. For example, early work demonstrated that as cells reach confluency, HSPG production is ramped up, nuclear HS levels increase 3-fold, and the structural motifs of HS in the nucleus change (Ferdarko and Conrad, 1986). The nuclear fraction of HS contains high amounts of 2-O sulfated glucuronic acid, and as the cells transition from logarithmic growth to confluency, there is a higher degree of sulfation within the nucleus. Therefore, increased levels of sulfation may decrease cell proliferation. The addition of exogenous HS to hepatoma cells causes cell division arrest in the G1 phase (Ferdarko et al., 1989). As nuclear levels of HS decrease, the cell cycle progresses through S, G2, and M phases. Therefore, cells undergoing mitosis lose nuclear HS, but after mitosis, the HS reappears in the nucleus (Stewart and Sanderson, 2014). In mesothelioma, the nuclear translocation of the HSPG syndecan-1 was linked to specific points of the cell cycle through interactions with microtubule structures (Brockstedt et al., 2002; Dobra et al., 2003), and drug-induced cell division arrest in the G2 phase inhibited the HSPG syndecan-1 translocation to the nucleus (Zong et al., 2009). Additionally, the distribution of the HSPG glypican in neurons was dynamic and changes correlated with different phases of the cell cycle (Liang et al., 1997). These data demonstrate a clear correlation between the cell cycle stage and nuclear localization of HSPGs.

In addition, recent studies (Shaberg et al., 2021) demonstrate that the sulfation of GAGs modulates the cell cycle of embryonic mouse spinal cord neural stem cells. Whether changes in HS sulfation, HSPGs, and/or HS GAGs in the nucleus are important initiating factors for cell cycle changes in AD and related tauopathies is an important topic for future studies.

### “AD-in-a-Dish”—What Causes Spontaneous Formation of Plaques, Then Tangles? HSPGs Are Key

Choi et al. (2014) and then Kim et al. (2015) turned the AD research world on its head by reproducing the formation of amyloid plaques first, followed weeks later by deposition of tangles, in a cell culture dish. This was the first recapitulation of both plaques and tangles in a 3D human neural cell culture system and they excitingly demonstrated that plaques come first, followed by tangles. When they blocked plaque formation, then tangles never formed. Human neural progenitor cells that produce high levels of Aβ were overexpressed with human beta-amyloid precursor protein (APP) or APP + presenilin 1 containing FAD mutations. The key to this model was they used “Matrigel” (BD Biosciences) as a “3D support matrix because it contains high levels of extracellular matrix proteins”. In 6-weeks, amyloid plaque formation spontaneously occurred, followed in 1–2 weeks later by the formation of neurofibrillary tangles (Choi et al., 2014). This probably worked because “Matrigel” contains the same perlecan (isolated from EHS sarcoma) that Castillo and Snow (2006) demonstrated formed maltese-cross spherical amyloid core plaques spontaneously following Aβ 1–40 + perlecan, or Aβ 1–40 + HS GAGs as shown in Figure 8 (Castillo and Snow, 2006). Choi et al. (2014) also added the highly sulfated GAG heparin to their cultures. Thus, both HSPGs from “Matrigel”, and highly sulfated GAGs from heparin probably contributed greatly to the spontaneous formation of Aβ plaques, and then tau tangles. It would be important to determine whether “AD-in-a-dish” still works to spontaneously form plaques, and then tangles, if “Matrigel” and/or heparin were not used as the underlying scaffold.

### HSPGs Are Produced by Microglia and Astrocytes and Play an Important Role in Inflammation

In a further modification study to the “AD-in-a-dish” model microglia and astrocytes were added to help further understand the role of inflammation in AD pathogenesis (Park et al., 2018). A similar use of “Matrigel” (containing large amounts of perlecan, a specific HSPG) and heparin was added to the cell culture media. Using this model of AD, microglia demonstrated a marked elevation of different inflammatory cytokines including IL-1, IL-6, IL-8, and TNF-α. Recruited microglia induced neuronal loss similar to a mechanism believed to occur in AD. In fact, besides the accumulation of plaques and tangles in the AD brain, many hypothesize the inflammation is the key event that drives AD pathology into clinical manifestations of severe memory loss and dementia. There are cases at autopsy that have brains with lots of plaques and tangles throughout, but these patients never showed clinical symptoms of memory loss or dementia (Mufson et al., 2016). The missing piece besides brain plaques and tangles is inflammation that drives the disease, neuronal loss, and eventual memory loss leading to dementia. HSPGs in the brain are produced by both microglia (Miller et al., 1997) and astrocytes (Ard and Bunge, 1988; Snow et al., 1988a; Snow and Wight, 1989)—and “HSPG dumping” into the extracellular matrix and the cells may occur in AD (O’Callaghan et al., 2018). Both ameboid and ramified microglia produce HSPGs (Miller et al., 1997). Perlecan and other HSPGs are produced by endothelial cells in the walls of blood vessels and contribute to cerebrovascular amyloid deposition in AD (Snow and Wight, 1989; Koyama et al., 1998; Van Horssen, 2001; Kinsella et al., 2003; Hosono-Fukao et al., 2012). In models of brain injury, the HSPGs glypican-1, syndecan-1, and syndecan-3 are upregulated in glial cells to participate in neurite outgrowth regulation (Iseki et al., 2002; Hagino et al., 2003a,b).

### Heparanases, Sulfatases, and Ext1 HS Genes: Key Modulators of HS GAG Production and Its Potential Importance in AD

Heparanase is an endo-β-glucuronidase and the only known mammalian glycosidase capable of cleaving HS GAG chains. HS cleavage by heparanase occurs at a limited number of HS-oligosaccharide fragments that are 10–20 saccharides in length (Zhang et al., 2014). Heparanase also plays a significant role in inflammation cleaving cell surface HS disrupting interactions with chemokines and selectins that recruit leukocytes (Merovitz et al., 2013). Transgenic mice that overexpress human heparanase produced drastic shortening of HS GAG chains in the liver and kidney and show a marked reduction of HS GAG and AA amyloid deposition in the liver and kidney in an experimental mouse model of AA amyloidosis (i.e., AEF + silver nitrate; Li et al., 2005). The spleen which escaped excessive heparanase effects retained almost full size HS GAG chains and remained susceptible to AA amyloid fibril deposition in that organ (Li et al., 2005). These studies indicated that a minimal chain length of HS GAGs is a prerequisite for efficient amyloid fibril formation and deposition *in vivo*. Agents such as small molecule sulfates and sulfonates (135–1,000 MW) have been postulated to be effective anti-amyloid compounds *in vitro* and *in vivo* (Kisilevsky et al., 1995, 2004).

Heparanases are upregulated in the brains of AD patients and immunolocalized to tangles in neurons and in parts of plaques, but mostly as a consequence (not the cause) of the disease in order to reduce the existing AD pathology by degrading HS chains (Garcia et al., 2017).

Sulfatases are extracellular endosulfatases that catalyze the 6-O-desulfation of HS GAGs (Dhoot et al., 2001). Treatment of AD brain sections and in older 18-month Tg 2576 transgenic mouse brain with sulfatase-1 or sulfatase-2 further demonstrated the presence of HS GAGs in the pathological lesions of AD (Hosono-Fukao et al., 2012). Sulfatase 2 (but not sulfatase 1) expression was decreased in AD transgenic mice, and in the hippocampus and frontal lobe of AD patients (Roberts et al., 2017) that suggests that enhanced sulfation of HSPGs may be a key contributing factor.

Ext1 gene encodes an essential glycosyltransferase for HS GAG biosynthesis. Removal of neuronal HS by conditional deletion of the Ex1 gene in postnatal neurons of APP/PS1 transgenic mice led to a reduction (by over 60%) in both Aβ oligomerization and the deposition of brain amyloid plaques in the hippocampus and cortex, without affecting APP processing (Liu et al., 2016). Activation of astrocytes and GFAP immunostaining was also markedly reduced in Ext1 knockout mice. Neuronal HS deficiency also resulted in the reduction of proinflammatory cytokines including TNF-α, IL-1, and IL-6 (Liu et al., 2016). *In vivo* microdialysis studies also detected an accelerated rate of Aβ clearance in the brain interstitial fluid indicating that increased neuronal HS either inhibited or represented an inefficient pathway for Aβ clearance (Liu et al., 2016).

### HS GAG Accumulation in Neurons as Observed in Mucopolysaccharidosis Diseases Lead to Misfolded Protein Accumulation Including Aβ, Tau Protein, α-Synuclein, and Prion Protein (PrP)

Mucopolysaccharidoses (examples Hunter’s syndrome—MPS II and Hurler’s syndrome; MPS I) are lysosomal storage diseases that are inborn errors in the metabolism of GAGs. Missing from birth are specific lysosomal enzymes needed to normally degrade HS [(examples lysosomal heparan-N-sulfatase and α-L-iduronidase in MPS I, iduronidate-2-sulfatase in MPS II; α-N-acetylglucosaminidase (NAGLU) and sulfamidase in MPS III)] and other GAGs leading to their massive accumulation in neurons and axons (Shapiro et al., 2017; Bigger et al., 2018). Symptoms of mucopolysaccharidoses include delayed development, language difficulties, sleep disturbance, loss of learned skills, motor and cognitive deficits in infants and children, many times leading to dementia and death before adulthood (Neufeld and Muenzer, 2001; Pshezhetsky, 2016; Aoyagi-Scharber et al., 2017; Zhou et al., 2020). This is a perfect human model to study the effects of early HS GAG accumulation in neurons that precedes any protein misfolding, as well as dysfunction of the autophagy-lysosomal pathway (ALP). In early studies (Ginsberg et al., 1999) increased HS GAGs in neurons was shown to demonstrate immunostaining for Aβ 1–40 in neurons throughout the brain in young patients with Hurler’s and Sanfilippo syndrome (MPS III). Later studies indicated that immunostaining in a Sanfilippo mouse model demonstrated increased accumulation of not only Aβ, but also tau protein, α-synuclein, and ubiquitin as early as 3 weeks, leading to axonal dystrophy (Beard et al., 2017). There was also massive neurodegeneration (>30%) and increasing levels of activated microglia and astrocytes throughout the brain with increased expression of inflammatory markers (Pshezhetsky, 2016). Marked increases of mitochondrial dysfunction were also observed by electron microscopy (Pshezhetsky, 2016). In another mouse model of MPS III, Thioflavin S positive deposits in neurons and axons were detected by antibodies to Aβ, tau, α-synuclein, and PrP further demonstrating that lack of HS GAG degradation in an initiating event that leads to misfolded proteins (Monaco et al., 2020). Exciting new treatments for mucopolysaccharidoses include a modified recombinant NAGLU that results in complete clearance of pathological HS and normalization of lysosomal storage pathology in a mouse model of Sanfilippo syndrome type B (Aoyagi-Scharber et al., 2017). CRL01 (daily injections) markedly reduced the pathology and neuroinflammation in a mouse model of MPS III (Monaco et al., 2020). These should be tested in transgenic mouse models of AD, and then in humans as possible new innovative treatments to decrease the pathology of AD by targeting HS GAG accumulation as an initiating event.

### Decreased Binding of ApoE to HSPGs Wards Off Familial Alzheimer’s Disease

Cell surface HSPGs are the receptors on the cell surface for Apolipoprotein E (ApoE) and the retention of ApoE4 and ApoE3 alleles depend on that interaction (Ji et al., 1998; Futamara et al., 2005; Saito et al., 2013). Whereas ApoE3 has a cysteine in position 112 and arginine at position 158, ApoE2 has a cysteine at both positions, and ApoE4 has an arginine. The ApoE4 allele is associated with a greater risk of developing AD, whereas the ApoE3 and ApoE2 alleles may be protective (Corder et al., 1993). ApoE4 shows about a 3-fold higher binding affinity to HSPGs than ApoE2 or ApoE3, suggesting that the ApoE4 allele that is a risk factor for AD binds tighter to HSPGs (Yamauchi et al., 2008; Fu et al., 2016).

This has been confirmed when a presenilin 1 mutation carrier from the largest autosomal dominant AD kindred was discovered not to develop mild cognitive impairment until her 70s, three decades after the expected age of onset. She had two copies of an ApoE3 Christchurch (ApoE3ch) mutation that reduced her ApoE binding to HSPGs (Arboleda-Velasquez et al., 2019). She had a high Aβ amyloid load in the brain by PET imaging, but a low tangle burden and neurodegeneration. Studies suggest that the ApoE3ch mutation that exerts a lower binding affinity to HSPGs is a potential area of further research, and once again places HSPGs at the forefront of AD pathogenesis.

### Bacteria and Viruses (Even SARS-CoV-2) All Use Cell Surface HSPGs to Enter Cells

Bacteria and viruses both use cell surface HSPGs to enter cells (Garcia et al., 2014, 2016). *Streptococcus pneumoniae* and bacteria involved in influenza, chlamydia, tuberculosis, Lyme disease, cystic fibrosis, meningitis, gonorrhea, gastrointestinal tract infections, pharyngitis, and pneumonia all use HS GAGs as receptors to enter cells (Fleckenstein et al., 2002; Baron et al., 2007; Garcia et al., 2014, 2016). Bacterial pathogens that enter the brain also use GAG binding to enter the central nervous system (Chang et al., 2011).

HS GAGs are also implicated in gingivitis and adult periodontitis (Embery et al., 1979; Rahemtulla, 1992), as gum disease is also postulated to be linked to cause AD. Specific HS GAG sulfatases are found in the human gut biome and thus HS GAG sulfatases are involved in bacteria of the gut as well (Ulmer et al., 2014). Improper breakdown of gut bacteria and microbes by lack of HS sulfatases may be a problem in AD (Cartmell et al., 2017). HS-modifying bacteria in human microbial communities may regulate viral adhesion, and loss of these commensals could predispose individuals to infections (Martino et al., 2020).

Viruses bind to cell surface HSPGs and HS GAGs on the cell surface to enter cells (Byrnes and Griffin, 1998; Liu and Thorp, 2001; Zhu et al., 2011; Khanna et al., 2017). This has been shown with Sindbis virus (Byrnes and Griffin, 1998), cytomegalovirus (Pitt et al., 2016), dengue virus, herpes simplex virus (Shukla and Spear, 2001; O’Donnell and Shukla, 2008; Hadigal et al., 2015; Vanheule et al., 2016), HIV (Connell and Lortat-Jacob, 2013), Akabane and Schallenberg virus (Murakami et al., 2017), Ebola virus (Tamhankar et al., 2018) and recently SARS-CoV-2 (Tavassoly et al., 2002; Clausen et al., 2020; Zhang et al., 2020), to name a few. Angiotensin converting enzyme 2 (ACE2) and cellular HS were both necessary for the binding of the SARS-CoV-2 spike protein ectodomain to enter cells (Tavassoly et al., 2002; Clausen et al., 2020; Zhang et al., 2020). The rapid Aβ fibril formation and response following virus infections (Elmer et al., 2018) is likely initiated first by an HS GAG response that then causes the formation of Aβ fibrils observed. Highly sulfated GAGs accelerate Aβ fibril formation within minutes (Castillo and Snow, 2006) and that is probably the initiating response to a bacterial or viral infection as well.

### The Anti-microbial Peptide Hypothesis of Alzheimer’s: Is it a Defense Mechanism or a HSPG/HS GAG Response?

A number of studies have suggested that Aβ may be a microbial peptide since bacteria and yeast form Aβ fibrils that appear to entrap such invaders by spewing amyloid fibrils that are Thioflavin S positive indicative of amyloid fibril formation (Kumar et al., 2016; Li et al., 2018; Muir et al., 2018). Further to this theory, the heparin-binding activity of “soluble Aβ oligomers appears to mediate targeting of microbial cell wall carbohydrates” (Muir et al., 2018).

We however postulate that HSPGs and/or HS GAG accumulation is an initial response to invading bacteria or viruses that directly lead to the acceleration and fibrillization of Aβ. It is the binding of bacteria or viruses to HS GAGs (not Aβ) that initiates this cascade response. This is not an entrapment of Aβ fibrils as a defense mechanism to invading bacteria or viruses but is likely an HSPG/HS GAG response that triggers Aβ aggregation to form fibrils nearly instantly (Castillo and Snow, 2006). HSPGs and HS GAGs are usually hard to observe and visualize at the electron microscopic (EM) level since they are usually washed out during EM processing. Cationic dyes such as ruthenium red or Cuprolinic blue such as shown in Figure 5 are needed to visualize PGs at the EM level. Thus, these authors have not proven that an increased HSPG or HS GAG response is not the initiating response prior to any Aβ fibril deposition. Until these studies are implemented one cannot rule out that Aβ fibril formation is actually formed following an HSPG and/or HS GAG response. Infections in APP transgenic mice by bacteria or viruses probably trigger enhanced HSPG and/or HS GAG deposition prior to Aβ fibril formation and deposition in tissues as shown in young Down’s syndrome brain (as early as 1 day after birth; Snow et al., 1990b) and in mucopolysaccharidosis (Ginsberg et al., 1999; Beard et al., 2017).

### Over-Sulfation of HSPGs in Alzheimer’s Disease (AD) Brain and During Brain Aging

Brain aging is a risk factor for AD and alters HS GAG accumulation by a variety of methods. Corpora amylacea, which are spherical bodies (10–100 microns) increase in the brain during aging contain highly sulfated GAGs, heparan sulfate (Snow et al., 1988b), and keratan sulfate (Liu et al., 1987). HSPGs and/or HS GAGs may be altered during brain aging and in AD suggesting that these molecules should be more closely studied (Yamada et al., 2017; Raghunathan et al., 2018; Huynh et al., 2019). One way to change HS GAG accumulation in the brain is due to splice variants in different PG genes that occur during aging. Four perlecan (one HSPG implicated in AD pathogenesis) domain I splice variants in the AD brain have been discovered that predict an HSPG with four HS GAG chains (instead of three), that leads to a marked increase in HS GAGs and sulfation that can initiate AD pathogenesis. In addition in the AD brain, using unique and specific antibodies these perlecan splice variants were immunolocalized in neurons, amyloid plaques, and/or neurofibrillary tangles. This discovery was initially reported in two issued US Patents (Maresh and Snow, 2002, 2009). We intend to publish and report the significance of this important discovery in a separate publication, but it again identifies HSPG and HS GAG alterations as a major initiating factor in AD pathogenesis.

### The New Unifying Hypothesis of Alzheimer’s Disease: HSPGs and HS GAGs Are Key Once Again

**Figure 9:**
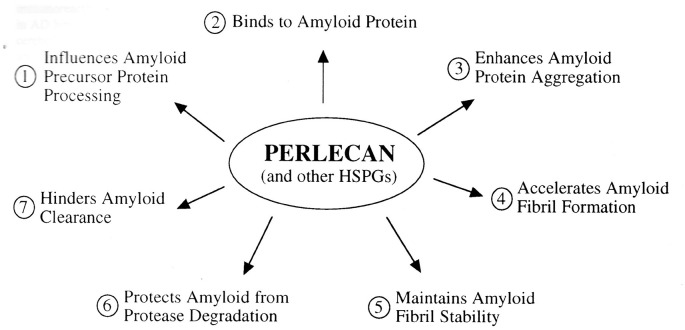
Perlecan and other HSPGs postulated in the pathogenesis of amyloidosis in general, and in AD in particular. The schematic outlines the various effects that perlecan and other HSPGs are postulated to play in the pathogenesis of amyloidosis in general and AD in particular. This was hypothesized by Snow and Wight in 1989. HSPGs were postulated to: (1) influence amyloid precursor protein processing; (2) bind to amyloid proteins; (3) cause an increased aggregation of amyloid proteins; (4) accelerate amyloid fibrils formation; (5) stabilize; (6) protect amyloid from protease degradation; and (7) inhibit amyloid clearance and removal. All of these have now been shown to be true (Snow and Wight, 1989).

**Figure 10:**
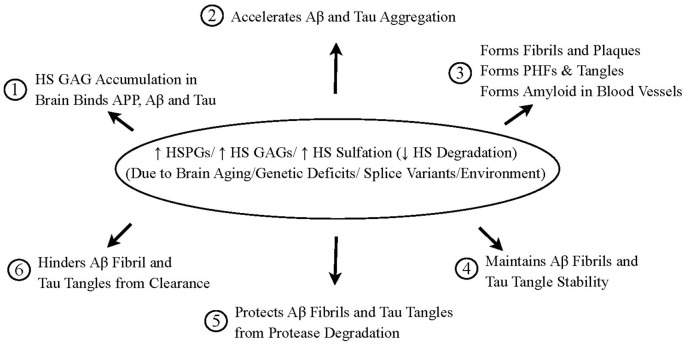
HSPGs and HS GAGs initiate and cause the brain pathology of AD. ↑ HSPGs (perlecan, agrin, glypicans, syndecans, undiscovered HSPGs)/ ↑ HS GAGs/ ↑ HS sulfation and/or ↓ HS degradation in the brain (in neurons, microglia, astrocytes, and/or endothelial cells) is postulated to occur due to: (a) brain aging, (b) genetic deficits; (c) splice variants; and/or (d) environment. All of these possibilities lead to increased HS GAGs and/or increased sulfation of HS GAGs. This then leads to: (1) increased binding to beta-amyloid precursor protein (APP), beta-amyloid protein (Aβ), and tau protein; (2) acceleration of Aβ and tau protein aggregation; (3) formation of Aβ amyloid fibrils, spherical maltese-cross amyloid plaques, and neuritic plaques; formation of paired helical filaments and tangles; and formation of Aβ amyloid deposits in blood vessel walls; (4) maintenance of Aβ amyloid fibril and tau tangle stability; (5) protection of Aβ fibrils and tau tangles from protease degradation; and (6) hindrance and blocking of the clearance of Aβ fibrils and tau protein tangles from the brain. This leads to the formation and persistence of brain plaques, tangles, and cerebrovascular amyloid, as well as increasing neuroinflammation all causative for the observed pathology in the AD brain. AD should be examined and tested to form new therapeutics like a mucopolysaccharidosis disorder since similar consequences (i.e., formation of congophilic and Thioflavin S-positive neuronal inclusions; increased deposition of Aβ; increased neuroinflammation; cognitive decline and eventual death) have all been observed in young children inheriting the lack of specific HS GAG degrading enzymes which usually leads to accelerated cognitive decline and early death.

Snow and Wight in 1989 first hypothesized that perlecan (and other HSPGs) are central to initiate AD pathogenesis (Figure 9). Much of this initial hypothesis has been proven over the last 30 years. The new Heparan Sulfate Proteoglycan Hypothesis of AD is shown in Figure 10. Increased HSPGs (including perlecan, agrin, glypicans, syndecans, and/or undiscovered HSPGs), increased HS GAGs, increased HS sulfation and/or decreased HS degradation in the brain (in neurons, microglia, astrocytes, and/or endothelial cells) are postulated to occur due to: (a) brain aging; (b) genetic deficits (ApoE4); (c) splice variants (perlecan domain I variants; other yet undiscovered splice variants); and/or (d) environmental factors, that may all contribute to AD pathogenesis: (1) HS GAG accumulation in brain binds to APP, Aβ, and tau protein; (2) HS GAGs and increased sulfation of GAGs accelerate Aβ and tau protein aggregation; (3) This induces Aβ to form fibrils and amyloid plaques, tau protein to form paired helical filaments and tangles, Aβ to form fibrils in the walls of blood vessels; (4) HS GAGs maintain Aβ fibril and tau tangle stability; (5) HS GAGs protect Aβ fibrils and tau tangles from protease degradation; and (6) HS GAGs hinder Aβ fibrils and tau tangles from clearance in brain. This unifying hypothesis accounts for most, if not all, AD research observations, including how plaques and tangles are formed. These will be important targets in AD research in the years to come.

## Author Contributions

AS wrote the manuscript and contributed to the planning, execution, writing, and final drafting of the manuscript. JC and TL edited the final manuscript for publication. All authors contributed to the article and approved the submitted version.

## Conflict of Interest

All authors were employed by company Cognitive Clarity Inc.

## Publisher’s Note

All claims expressed in this article are solely those of the authors and do not necessarily represent those of their affiliated organizations, or those of the publisher, the editors and the reviewers. Any product that may be evaluated in this article, or claim that may be made by its manufacturer, is not guaranteed or endorsed by the publisher.
